# To Line Artificial Dentures with Aluminum

**Published:** 1904-09

**Authors:** Clint W. La Salle

**Affiliations:** Rochester, N. Y.


(Written for *The American Journal of Dental Science.*)

### TO LINE ARTIFICIAL DENTURES WITH ALUMINUM.

By Clint W. La Salle, D. D. S., Rochester, N. Y.

(Written for *The American Journal of Dental Science.*)

The articles needed are a portion of Aluminum dust such as is used by plumbers in bronzing iron work; also make a solution of one part chloroform, two parts naphtha, and two parts carbon disulphide.

Boil out the flask and pack as usual, being careful to remove all superfluous rubber, and be sure the flask comes together under gentle pressure only. In packing, it is best to make the palatal portion of the pack of as large pieces as possible, bringing the flask together with a dampened cover cloth, which will give a smooth surface, to which the lining is to be applied.

After this has been done, take a swab of cotton wound around the end of a slender stick, and dip in the solution as given above. Apply this dampened swab to the exposed surface of rubber in flask. Make another swab as before, wet it in the solution, dip in powder and apply to surface of rubber previously moistened. Repeat this procedure, using new swabs each time, until the rubber refuses to hold any more of the aluminum powder. Now take a new swab, dry, and dip it in the powder and apply to the model, rubbing in such as will adhere to the surface thereof. Bolt the flask together and vulcanize as usual.

After vulcanizing, the flask must be stone cold before opening, and a reasonable amount of care should be exercised in removing portions of plaster adherent to the palatal portions of the plate. This can be easily done by the use of a blunt stick of soft wood; following this by the use of a soft brush in soap suds will give the required finish.

These directions faithfully followed will produce a metal lining that is one with the plate, and one which will wear indefinitely. It is claimed for this lining that its chief value is prophylactic in character, in that the absolutely smooth surface obtained by its use prevents lodgment of mucous concretions, which ordinarily act as a breeding-ground for organic growth.

